# Mechanisms and Functions of Chromophore Regeneration in the Classical Visual Cycle: Implications for Retinal Disease Pathogenesis and Therapy

**DOI:** 10.3390/biom15121676

**Published:** 2025-12-01

**Authors:** Xinyue Yu, Hao Fan, Hui Zhang, Xiaorong Li

**Affiliations:** 1 Tianjin Key Laboratory of Retinal Functions and Diseases, Tianjin Branch of National Clinical Research Center for Ocular Disease, Eye Institute and School of Optometry, Tianjin Medical University Eye Hospital, Tianjin 300384, China; xinyuera@tmu.edu.cn; 2 Tianjin Third Central Hospital, Tianjin 300384, China; fh960316@tmu.edu.cn

**Keywords:** classical visual cycle, 11-cis-retinal, chromophore, visual perception, retinal disease

## Abstract

11-cis-retinal, the indispensable chromophore of photoreceptor opsins, is fundamental for light detection and the initiation of visual signal transduction. Its synthesis and regeneration through the visual cycle are critical not only for phototransduction but also for maintaining retinal homeostasis. Disruption of key enzymes, such as retinal pigment epithelium (RPE)65 and retinol dehydrogenases, results in toxic retinoid accumulation, oxidative stress, and progressive photoreceptor degeneration. These pathological mechanisms contribute to inherited and acquired retinal diseases, including Stargardt disease type 1, age-related macular degeneration, Leber congenital amaurosis, retinitis pigmentosa, and fundus albipunctatus. Recent therapeutic advances, ranging from gene replacement therapy with RPE65 (voretigene neparvovec, Luxturna^®^) to small-molecule modulators and antioxidant strategies, underscore the translational potential of targeting chromophore metabolism. This review outlines molecular processes underlying chromophore synthesis and regeneration, elucidates how disruptions in these processes contribute to inherited and acquired retinal pathologies, and evaluates existing and emerging therapeutic strategies that target chromophore metabolism. We highlight ongoing challenges and critical knowledge gaps to guide future investigations on basic science, translational research, and clinical practice. This review provides a comprehensive overview of the molecular mechanisms, current therapeutic approaches, and outstanding challenges, with a focus on future intervention directions.

## 1. Literature Search Strategy

To ensure a comprehensive and up-to-date synthesis of current knowledge, a systematic literature search was conducted in July 2025 using PubMed, Web of Science, Scopus, and Embase. The search covered publications from January 2010 to June 2025 and focused on advances in visual-cycle mechanisms, chromophore regeneration, retinal degenerative diseases, and emerging therapeutic approaches. A broad combination of keywords and MeSH terms was used, including visual cycle, chromophore regeneration, 11-cis-retinal, retinal pigment epithelium, retinal degeneration, gene therapy, small-molecule modulators, antioxidants, and disease-specific terms such as Stargardt disease type 1, age-related macular degeneration, retinitis pigmentosa, Leber congenital amaurosis, and fundus albipunctatus.

Studies were eligible for inclusion if they directly addressed mechanisms of visual-cycle regulation or dysregulation, or if they examined therapeutic strategies relevant to retinal disorders. Only peer-reviewed original research (including in vitro, in vivo, and clinical studies), systematic reviews, and meta-analyses published in English were considered. Research based on mammalian models—including mice, rats, and human subjects—was prioritized to align with the translational focus of the review, although non-human primate or cell-based studies were included when they provided essential mechanistic insight. Non-peer-reviewed materials, studies unrelated to visual-cycle biology or retinal diseases, and non-mammalian models without direct relevance were excluded.

## 2. Introduction

Vision is the principal modality of human perception and contributes to approximately 80% of all sensory information processing. In addition to enabling spatial navigation, object identification, and cognitive maturation, visual function profoundly influences the overall quality of life. According to estimates from the World Health Organization, more than 250 million individuals worldwide experience moderate-to-severe visual impairment, resulting in considerable healthcare and socioeconomic challenges [1]. Consequently, elucidating the molecular pathways underlying the maintenance of visual function is not only biologically significant but also clinically important.

Central to vertebrate vision is the canonical visual cycle, an evolutionarily conserved metabolic process responsible for regenerating 11-cis-retinal chromophores [2]. This chromophore forms a covalent bond with rod and cone visual pigments, thereby enabling photon absorption and initiating a phototransduction cascade. Moreover, 11-cis-retinal plays a vital role in the maintenance of retinal cellular homeostasis and metabolic integrity [3].

**Figure 1:**
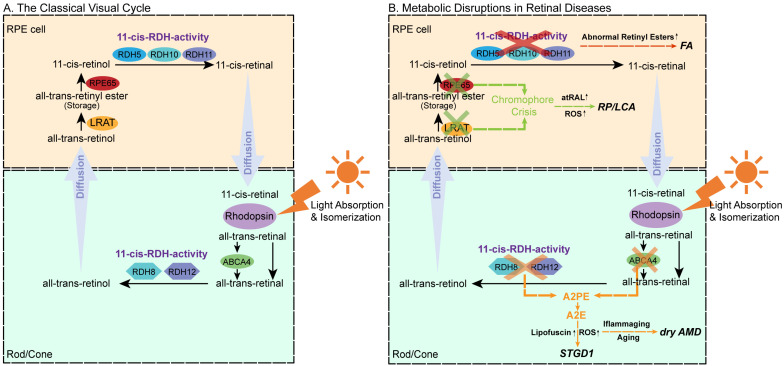
Schematic of the classical visual cycle, highlighting the key enzymes and transport proteins in the chromophore regeneration pathway and their relationship to various retinal diseases. (**A**) The classical visual cycle. (**B**) Metabolic disruptions in retinal diseases. Solid arrows indicate the normal metabolic flow, whereas dotted arrows represent pathological routes. “x” marks enzymes or transport proteins whose function is impaired or absent in specific disease conditions. Abbreviation explanation: LRAT, retinol: lecithin acyltransferase; PR65, RPE-specific 65 kDa protein, RDH5,11-cis-retinol dehydrogenase 5; RDH11, 11-cis-retinol dehydrogenase 11; RDH8, all-trans-retinol dehydrogenases 8; RDH12, all-trans-retinol dehydrogenases 12; ABCA4, ATP-binding cassette subfamily A member 4; A2E, *N*-retinyl-*N*-retinylidene ethanolamine; A2PE, *N*-retinylidene-*N*-retinyl-phosphatidylethanolamine; ROS, reactive oxygen species; atARL, all-trans-retinal; RP, Retinitis pigmentosa; LCA, Leber congenital amaurosis; FA, Fundus albipunctatus; AMD, Age-Related Macular Degeneration; STGD1, Stargardt disease type 1.

The regeneration of 11-cis-retinal relies on a tightly regulated network of enzymes and transport proteins, including retinal pigment epithelium (RPE)65, lecithin: retinol acyltransferase (LRAT), retinol dehydrogenases (RDHs), and ATP-binding cassette transporter A4 (ABCA4) [3,4] (Figure 1A). Dysregulation within this network disrupts chromophore homeostasis, leading to the accumulation of cytotoxic intermediates, notably all-trans-retinal (atRAL) and bis-retinoids, such as *N*-retinylidene-*N*-retinylethanolamine (A2E) [5]. These disturbances induce oxidative stress, lipid peroxidation, and, ultimately, photoreceptor apoptosis, thereby driving retinal degeneration.

Visual cycle dysfunction underlies a wide spectrum of inherited and acquired retinopathies. These include Stargardt disease type 1 (caused by mutations in ABCA4) [4,6], AMD—the leading cause of irreversible blindness in older adults, retinitis pigmentosa (RP), and Leber congenital amaurosis (LCA) (associated with mutations in LRAT, RPE65, and RDHs) [7], as well as fundus albipunctatus (FA) (linked to RDH5 and RLBP1 mutations) [8]. Despite their diverse genetic origins, these disorders converge in shared pathological cascades involving chromophore dysregulation (Figure 1B).

Over the past two decades, significant scientific advances have greatly expanded our understanding of the molecular pathways governing vision, while simultaneously accelerating the development of innovative therapies. Gene therapy has now entered clinical practice, with voretigene neparvovec (Luxturna^®^) for RPE65-mediated Leber congenital amaurosis representing a landmark clinical milestone [9,10]. Complementary pharmacological approaches, including small-molecule modulators, antioxidant agents, and chromophore supplementation, have been developed to restore metabolic homeostasis [11,12,13]. Strategies in cellular and regenerative medicine continue to broaden the spectrum of translational opportunities [14].

This review consolidates recent advancements to outline the molecular processes underlying chromophore synthesis and regeneration, elucidate how disruptions in these processes contribute to inherited and acquired retinal pathologies, and evaluate existing and emerging therapeutic strategies that target chromophore metabolism. Finally, we highlight the ongoing challenges and critical knowledge gaps in guiding future investigations in basic science, translational research, and clinical practice.

## 3. Molecular Mechanisms of the Visual Cycle

### 3.1. Precycle Events

In photoreceptor cells (rods and cones), vision is initiated when the chromophore 11-cis-retinal covalently binds to opsins, forming light-sensitive pigments, such as rhodopsin (λmax ≈ 498 nm) [15,16]. Upon photon absorption, 11-cis-retinal undergoes rapid cis-to-trans isomerization, generating atRAL, the primary trigger for phototransduction [17]. This conformational change activates opsin, initiating a G-protein-coupled signaling cascade that amplifies single-photon responses up to 10^5^-fold, ensuring high sensitivity under low-light conditions [18,19].

atRAL clearance occurs under strict metabolic regulation. Under physiological conditions, retina-specific retinol dehydrogenase (RDH)8 and RDH12 efficiently convert atRAL to all-trans-retinol (atROL), thereby maintaining retinaldehyde homeostasis [20]. Evidence from knockout models further underscores the critical roles of these enzymes. For instance, Rdh8^−/−^ mice exhibit a more than three-fold increase in atRAL levels after light exposure, whereas Rdh12^−/−^ mice develop progressive photoreceptor degeneration during prolonged illumination [21,22].

When the atRAL production exceeds its clearance capacity, a protective detoxification pathway is activated. ABCA4, an ATP-binding cassette transporter localized in photoreceptor disc membranes, cooperates with RDH12 to transport atRAL across the disc membrane [23,24]. ABCA4 deficiency, as demonstrated in Abca4^−/−^ mice, results in the accumulation of bisretinoids, such as A2E and lipofuscin, thereby recapitulating key features of the human Stargardt phenotype [23]. Thus, precycle events integrate photon capture with atRAL detoxification to establish a foundation for a functional and sustainable visual cycle.

### 3.2. Core Metabolic Phase

Once generated, atROL diffuses or is transported into the RPE, where it undergoes enzymatic recycling into 11-cis-retinal. The classical visual cycle proceeds through four tightly coordinated stages.

#### 3.2.1. Esterification

LRAT esterifies atROL with acyl-CoA, producing all-trans-retinyl esters (atRE) [25]. This reaction stabilizes retinol for storage and provides substrates for isomerization. LRAT^−/−^ mice exhibit a greater than 90% reduction in retinyl ester pools and congenital blindness, highlighting LRAT’s essential role [26].

#### 3.2.2. Isomerization–Hydrolysis

RPE65, a Fe^2+^-dependent isomerohydrolase, converts atRE into 11-cis-retinol (11cROL), and its activity constitutes the rate-limiting step of the cycle [27]. Rpe65^−^/^−^ mice fail to produce 11-cis-retinoids, exhibit absent rod function, and undergo progressive cone degeneration, recapitulating features of human LCA type 2 (LCA2) [28].

#### 3.2.3. Oxidation

RDH enzymes, notably RDH5, RDH10, and RDH11, catalyze the oxidation of 11cROL to 11-cis-retinal (11cRAL). Mutations in RDH5 in humans cause fundus albipunctatus, a stationary night blindness disorder characterized by delayed dark adaptation [29,30].

#### 3.2.4. Trafficking and Delivery

Intracellular binding proteins mediate precise retinoid distribution. CRALBP (RLBP1) binds to 11cRAL with a sub-nanomolar affinity, shielding it from nonspecific reactions and facilitating opsin reconstitution, whereas CRBP1 (RBP1) regulates retinol trafficking [31]. Mutations in RLBP1 lead to retinitis punctata albescens and Bothnia dystrophy, highlighting the critical role of retinoid chaperones [32].

Through this coordinated process, the RPE successfully sustains the regeneration of 11-cis-retinal, which is essential for ongoing visual function. Consequently, any impairment within this pathway, whether due to LRAT, RPE65, RDH, or RLBP1 deficiency, disrupts chromophore production and initiates pathological cascades, culminating in retinal degeneration.

### 3.3. Protective and Regulatory Mechanisms

The visual cycle, which forms the metabolic basis of phototransduction, operates in conjunction with dedicated protective mechanisms that preserve retinal integrity under varying light exposures and oxidative stress.

#### 3.3.1. Photon Capture and Signal Amplification

The combination of rhodopsin’s high quantum efficiency (~0.67) and the substantial signal amplification through the G-protein cascade ensures that even single-photon events are detectable [18]. However, this high efficiency renders the system susceptible to retinoid overloading under light stress. Therefore, robust detoxification mechanisms are required.

#### 3.3.2. Antioxidant Defense

The accumulation of excess atRAL or bisretinoids stimulates ROS production. Endogenous antioxidant defenses, including superoxide dismutase, catalase, and glutathione peroxidase, are activated [33]. The critical importance of maintaining redox balance is demonstrated in Abca4^−/−^ models, where administration of exogenous antioxidants, such as quercetin and *N*-acetylcysteine, attenuates lipofuscin formation and preserves photoreceptor viability [34,35].

#### 3.3.3. Metabolic Feedback Regulation

Precise tuning of retinoid flux involves coordinated feedback at multiple levels. The gene expression of enzymes, such as LRAT and RPE65, is modulated by retinoic acid signaling [36,37]. In contrast, the activity of transporters, such as ABCA4, is adjusted according to substrate levels. Moreover, the visual cycle is linked to cellular stress pathways, as exemplified by the PERK-eIF2α axis, which regulates protein translation under oxidative stress [38,39].

Together, these mechanisms ensure that chromophore regeneration maintains a balance between efficiency and safety, effectively preventing the accumulation of cytotoxic byproducts and promoting lifelong retinal health.

The key kinetic parameters, substrates, and functional characteristics of major visual-cycle enzymes and transporters are summarized in Table 1.

## 4. Chromophore Metabolic Abnormalities and Retinal Diseases

### 4.1. Stargardt Disease Type 1 (STGD1)

STGD1 is the most common inherited juvenile macular dystrophy, predominantly caused by mutations in ABCA4 that impair the clearance of retinoid intermediates in photoreceptor outer segments [40]. This impairment results in the accumulation of bisretinoids, particularly A2E [41], within RPE cells, thereby inducing mitochondrial dysfunction, oxidative stress, and progressive photoreceptor degeneration [42].

#### 4.1.1. ABCA4 and RDH8 Synergistic Pathogenic Mechanisms

ABCA4, a photoreceptor-specific ATP-binding cassette transporter, normally exports A2PE from the disc lumen to the cytoplasmic leaflet for clearance [43]. Loss-of-function mutations in ABCA4 impair this process, leading to A2PE retention and its photochemical conversion into A2E. Excessive A2E within RPE cells disrupts mitochondrial and lysosomal integrity, contributes to Bruch’s membrane thickening, and ultimately results in central vision loss [23,44]. Molecular modeling further indicates that transporter dysfunction prolongs A2PE half-life and amplifies bisretinoid formation, imposing chronic metabolic stress on the RPE [45].

RDH8 (prRDH) is a key visual cycle enzyme that reduces atRAL to atROL while sparing 11-cis-retinal [46]. Rdh8^−/−^ mice appear morphologically normal under dim light but accumulate toxic atRAL under bright illumination, displaying delayed photoreceptor recovery [47]. Thus, RDH8 protects retinal integrity by accelerating atRAL clearance, maintaining rhodopsin homeostasis, and limiting ROS formation.

The combined deficiency of ABCA4 and RDH8 exacerbates bisretinoid accumulation and photoreceptor degeneration. In Abca4^−/−^ mice, RPE A2E content is up to 12-fold higher than in wild-type controls at 9 months [48]. Double-knockout Abca4^−/−^Rdh8^−/−^ mice exhibit earlier and more severe phenotypes, with toxic metabolite accumulation evident by 3 months [21,41]. Clinical evidence further supports this synergy; patients with combined ABCA4 and RDH8 mutations show faster disease progression, especially under high light exposure, where A2E accumulation rates increase up to six-fold compared to those with single mutations [49,50].

#### 4.1.2. Pharmacological and Gene-Based Therapeutic Strategies for STGD1

Therapeutic strategies for STGD1 primarily aim to reduce bisretinoid accumulation, alleviate oxidative stress, and correct genetic defects. Modulation of the visual cycle has been explored through the use of non-retinoid RPE65 inhibitors such as emixustat, which effectively lowers A2E accumulation in mouse models [51]. However, derivatives such as MB-002 fail to confer photoprotection due to weak atRAL binding and limited bioavailability [52,53,54]. This indicates that RPE65 inhibition alone is insufficient and that structural optimization and multi-targeted strategies are required.

Given the close association between atRAL cytotoxicity and ROS overproduction, antioxidant therapy has emerged as a complementary strategy [45]. Natural antioxidants, such as quercetin, have demonstrated efficacy; for example, Wu et al. observed that quercetin restores the viability of atRAL-exposed 661 W photoreceptor cells to 70% of control levels while simultaneously reducing ROS [38]. In Abca4^−/−^Rdh8^−/−^ mice, quercetin treatment suppressed PERK signaling, mitigated outer nuclear layer thinning by 38%, and improved visual performance in behavioral tests [38]. These results highlight the potential of quercetin as a multi-mechanistic agent that mitigates oxidative injury and modulates apoptotic pathways.

In addition to small molecules, gene therapy has achieved notable progress in monogenic retinopathies, such as STGD1, primarily using AAV vectors [55,56,57]. However, AAV-based delivery of ABCA4 is limited by the large size of the gene and host immune responses, prompting the exploration of dual-vector systems and lentiviral alternatives, which show promise in preclinical studies [58]. CRISPR-based gene editing, while having considerable potential, faces challenges related to safe and efficient in vivo delivery and target specificity [36].

In summary, the current research highlights critical directions for future development: optimizing delivery systems (e.g., for tropism and packaging capacity), designing combination regimens that integrate gene therapy with pharmacological agents, establishing scalable production and standardized protocols, and conducting comprehensive long-term safety assessments. Addressing these priorities is essential for translating next-generation therapies for retinal degeneration into clinical practice.

### 4.2. Age-Related Macular Degeneration (AMD)

AMD is the leading cause of irreversible central vision loss in the elderly and arises from the complex interplay of genetic predisposition, oxidative stress, and impaired visual cycle activity [59,60]. Core pathological features include RPE dysfunction, Bruch’s membrane thickening, and drusen formation, all of which disrupt photoreceptor–RPE metabolic exchange and promote chronic inflammation [61,62].

#### 4.2.1. Visual Cycle Inefficiency and Retinoid Toxicity

With aging, the efficiency of RPE65-mediated 11cRAL regeneration declines, resulting in excess atRAL accumulation and ROS generation [63]. This retinoid imbalance parallels the toxic stress observed in ABCA4-related STGD1, though it arises from aging and environmental influences rather than monogenic mutation. Persistent atRAL reacts with phosphatidylethanolamine to form A2E, which accumulates in RPE cells and drives progressive lipofuscinogenesis [64].

Recent evidence further indicates that this oxidative burden promotes ferroptosis, an iron-dependent form of programmed cell death characterized by lipid peroxidation and mitochondrial dysfunction [65]. In the aging or metabolically stressed retina, iron overload and compromised antioxidant defenses sensitize RPE and photoreceptor cells to ferroptotic injury [66,67].

#### 4.2.2. Pharmacological and Biophysical Therapeutic Strategies for Dry AMD

Apocarotenoids, such as 9′-cis-norbixin (BIO201), exhibit dual protective mechanisms relevant to AMD pathology. In vitro studies show that BIO201 protects RPE cells from blue light- and A2E-induced damage, while in vivo experiments demonstrate preserved visual function in AMD animal models [68,69]. Its optimized derivative, BIO203, shows enhanced therapeutic potential via several key improvements: (i) structural modifications enhance ocular bioavailability and pharmacokinetics; (ii) it suppresses A2E-induced inflammatory markers (IL-6, IL-8) and VEGF; and (iii) prolonged oral administration over 6 months maintains retinal structure and electrophysiological function in AMD models (blue light-exposed rats and Abca4^−/−^Rdh8^−/−^ mice) [70].

Given the central role of chronic, age-related low-grade inflammation (“inflammaging”) in dry AMD pathogenesis [71,72], these anti-inflammatory and antioxidant actions provide a strong mechanistic basis for BIO203-mediated neuroprotection. By counteracting inflammaging-associated cytokine dysregulation and oxidative stress, BIO203 supports both photoreceptor preservation and long-term retinal homeostasis in age-related retinal degeneration [70]. Collectively, these findings establish BIO203 as a promising preclinical candidate for the management of dry AMD and other inflammatory retinal diseases.

Emerging evidence also implicates the hepcidin–ferroportin axis in age-related retinal iron accumulation and subsequent ferroptosis [73]. Under physiological conditions, the hormone hepcidin limits intracellular free iron by binding to the iron-export protein ferroportin and promoting its internalization and degradation. In the aging retina and in experimental AMD models, dysregulation of this pathway leads to excessive iron retention, oxidative lipid damage, and heightened susceptibility of RPE and photoreceptor cells to ferroptotic death [74]. Although clinical trials of hepcidin agonists or mimetics in AMD have not yet been reported, therapeutic modulation of hepcidin signaling—or mimicking its iron-restrictive effects—represents a novel strategy to reduce retinal free iron, mitigate iron-driven ROS formation and lipid peroxidation, and inhibit ferroptosis. Such interventions could complement the antioxidant and anti-inflammatory effects of agents like BIO203 and contribute to a multimodal therapeutic framework for dry AMD.

In addition to pharmacological approaches, non-invasive biophysical interventions such as photobiomodulation (PBM) have gained attention for their neuroprotective potential [75,76]. PBM uses extremely low-irradiance red-to-near-infrared light to enhance mitochondrial respiratory efficiency, reduce oxidative stress, and modulate inflammatory signaling within retinal cells. Clinical evidence indicates that short-term, low-irradiance PBM significantly improves retinal function in patients with dry AMD [77]. These results suggest that PBM may serve as a valuable adjunct to emerging pharmacologic and genetic therapies by safely and repeatedly improving retinal bioenergetics.

### 4.3. Retinitis Pigmentosa (RP) and Leber Congenital Amaurosis (LCA)

RP and LCA are prototypical inherited retinal dystrophies characterized by progressive dysfunction and degeneration of the photoreceptors and RPE. Globally, RP affects approximately 1 in 3500–7000 individuals and is clinically defined by a triad of features, including early onset night blindness, progressive visual field constriction leading to late-stage tubular vision, and characteristic bone-spicule pigment deposits in the fundus, ultimately culminating in irreversible blindness [78]. In contrast, LCA represents the most severe form of inherited retinal degeneration, accounting for 10–20% of childhood blindness of genetic origin. Affected infants typically present with profound visual impairment within the first few months of life and exhibit a non-recordable (“flat”) electroretinogram [79].

A central pathogenic mechanism common to both RP and LCA involves disruption of the visual cycle, particularly the toxic cascade triggered by abnormal atRAL metabolism and accumulation [80]. Excess atRAL induces ROS overproduction, driving oxidative stress that promotes lipid peroxidation, mitochondrial dysfunction, and DNA fragmentation, ultimately leading to photoreceptor apoptosis [81].

#### 4.3.1. LRAT and RPE65 Synergistic Pathogenic Mechanisms

LRAT and RPE65 are two indispensable enzymes in the classical visual cycle, acting sequentially within the RPE to regenerate the visual chromophore 11cRAL [82]. LRAT catalyzes the esterification of atROL into all-trans-retinyl esters, thereby establishing the essential substrate pool for RPE65. Subsequently, RPE65 converts these retinyl esters into 11cROL through an Fe^2+^-dependent isomerohydrolase reaction [83]. This tightly coordinated process maintains a continuous chromophore supply, ensuring efficient rhodopsin regeneration and preserving photoreceptor sensitivity after light exposure.

Pathogenic variants in either enzyme interrupt the visual cycle at distinct yet interdependent steps, leading to a “chromophore crisis” that ultimately drives photoreceptor degeneration. Pathogenic LRAT mutations reduce retinyl ester synthesis by over 70% and deplete RPE ester stores by approximately 90%, causing profound 11cRAL deficiency and combined rod–cone dysfunction [84,85]. Lrat^−/−^ mice display shortened outer segments, elevated ERG thresholds, and progressive photoreceptor apoptosis, consistent with severe visual decline [86,87]. In contrast, RPE65 deficiency abolishes 11cRAL production, completely blocking the visual cycle. Rpe65^−/−^ mice exhibit profound rod dysfunction, while cone photoreceptors degenerate rapidly from birth, rendering the retina nearly light-insensitive [88,89]. Although cones constitute only ~3% of murine photoreceptors, their early loss highlights the heightened vulnerability of cone metabolism to chromophore deprivation [90].

The interdependence of LRAT and RPE65 is further illustrated by their synergistic pathogenic effects: LRAT deficiency deprives RPE65 of its substrate, whereas RPE65 loss causes retinyl ester accumulation, disrupting retinoid homeostasis [91]. Both defects promote secondary oxidative stress through atRAL buildup, increased ROS generation, and mitochondrial impairment. Moreover, deficiencies in additional retinal dehydrogenases, such as RDH12, intensify these disturbances by further amplifying toxic metabolite accumulation and accelerating photoreceptor apoptosis [81,92].

Clinically, complete loss-of-function mutations in LRAT or RPE65 result in LCA, whereas hypomorphic variants produce later-onset RP [93,94]. This phenotypic spectrum reflects both the shared biochemical pathway linking these enzymes and the distinct consequences of specific mutational severities.

#### 4.3.2. Gene-Based and Pharmacological Therapeutic Advances for RP and LCA

Targeted therapeutic strategies for visual cycle abnormalities have advanced significantly by providing molecular insights into dysregulation of retinoid metabolism. The landmark approval of Luxturna^®^ (voretigene neparvovec) by the U.S. Food and Drug Administration (FDA) in 2017 established gene therapy as a paradigm-shifting intervention for RPE65-associated LCA2 [82,93,95,96]. Phase III clinical trials have demonstrated that bilateral subretinal delivery of AAV2-RPE65 restored ERG amplitudes to approximately 65% of baseline values, reduced dark-adaptation thresholds by 3.2 log units, and sustained functional vision improvement for at least 4 years in 31 genotypically confirmed patients, with no serious adverse events at 1-year follow-up and durable efficacy across 3–4 years of monitoring [97,98,99]. This milestone establishes a proof-of-concept that gene therapy can correct visual cycle defects in humans.

Parallel innovations have emerged. For example, China’s LX101 injection has received a breakthrough therapy designation, whereas CRISPR-Cas9-mediated correction of Rpe65 mutations in murine models delayed photoreceptor degeneration by approximately 40% through precise genomic editing [36]. In addition to genetic approaches, small-molecule modulators have also been developed. MB-001, a competitive RPE65 inhibitor that binds the Fe^2+^-coordinating active site, demonstrated neuroprotection in preclinical models: it preserved ~40% of outer nuclear layer thickness in light-damage paradigms. It reduced ERG amplitude decline by 53% (*p* < 0.01) in RPE65-overexpressing RP models by suppressing aberrant atRAL isomerization [34].

Metabolic compensation represents a further expansion of therapeutic tools. The oral delivery of 9-cis-retinyl acetate offers a means to partially compensate for the deficits of 11cRAL, and combination strategies designed to address failures in multiple enzymatic pathways are under active investigation [100].

In summary, a crisis in chromophore homeostasis driven by dysregulated atRAL and the resulting oxidative stress lies at the heart of RP and LCA pathology. The established success of RPE65 gene replacement underscores the viability of targeting the visual cycle, whereas novel interventions involving small molecules, metabolic compensation, and gene editing have progressively widened the scope of treatment. Future progress is expected to hinge on the development of integrated combinatorial regimens precisely tailored to the specific genetic and metabolic profiles of each disorder.

### 4.4. Fundus Albipunctatus (FA)

FA is a rare autosomal recessive retinopathy that clinically presents as congenital stationary night blindness, characterized by markedly delayed dark adaptation (>30 min compared with physiological adaptation <10 min) [101]. A hallmark diagnostic feature is the presence of diffuse white dot deposits on fundus examination, which histologically corresponds to abnormal retinyl ester accumulation within the RPE. These deposits progressively interfere with retinal metabolism and structure, and in advanced disease, contribute to photoreceptor dystrophy and significant visual disabilities [102].

At the molecular level, FA most commonly results from mutations in RDH5 [103], which encodes an RPE-specific microsomal dehydrogenase that catalyzes the oxidation of 11-cis-retinol to 11-cis-retinal, an essential step in rhodopsin regeneration and dark adaptation [104,105]. Although isoenzymes, such as RDH10 and RDH11, provide limited residual activity (<20% of wild-type efficiency), their compensatory effect is insufficient to prevent disease progression [43,106].

#### 4.4.1. RDH5 and RLBP1 Synergistic Pathogenic Mechanisms

FA is primarily caused by mutations in RDH5 and, less frequently, RLBP1, both of which play interdependent roles in the regeneration of 11cRAL within the visual cycle. RDH5, localized in the RPE, catalyzes the oxidation of 11cROL to 11cRAL. Loss-of-function mutations in RDH5 lead to the accumulation of 11/13-cis-retinol, resulting in markedly delayed dark adaptation. The excess cis-retinol is aberrantly esterified by LRAT, generating atypical retinyl esters that disrupt retinoid flux and promote lipofuscin-like deposits [107,108]. Although Rdh5^−/−^ mice retain largely intact retinal architecture, recovery after light exposure is severely delayed, indicating that dysfunction arises from biochemical rather than structural impairment. Partial compensation by RDH11 provides limited detoxification but is insufficient to restore normal visual cycle kinetics [109].

RLBP1 acts downstream of RDH5 by stabilizing and shuttling 11-cis-retinoids. By tightly binding 11cRAL (Kd < 20 nM) and preventing product inhibition of RPE65, CRALBP supports chromophore regeneration and maintains steady retinoid turnover [110,111]. Mutations in RLBP1 disrupt ligand binding and produce a spectrum of phenotypes, including FA, retinitis punctata albescens, and Bothnia dystrophy [112,113]. In Rlbp1^−/−^ mice, 11cRAL regeneration is delayed by more than an order of magnitude and is accompanied by cone-dominant dysfunction, M-opsin mislocalization, and selective cone degeneration [114].

Mechanistically, deficiencies in RDH5 and RLBP1 exert synergistic pathogenic effects: loss of RDH5 limits substrate oxidation, whereas impaired CRALBP function reduces retinoid trafficking and stabilization. Together, these defects compromise chromophore regeneration and promote toxic retinyl ester accumulation in the RPE. This biochemical cascade provides a coherent explanation for the characteristic white-dot lesions and night blindness observed in FA.

#### 4.4.2. Gene, Metabolic, and Cellular Therapeutic Strategies for FA

Although no disease-modifying therapies for FA have received clinical approval, the emergence of pathogenesis-driven targeted strategies is encouraging. The present investigative efforts have concentrated on three complementary avenues: gene-based correction, metabolic compensation, and cellular replacement therapies.

Gene therapy remains the leading strategy for precise correction of pathogenic mutations. CRISPR/Cas9-mediated genome editing has the potential to repair RDH5 mutations and restore 11-cis-retinal synthesis directly. However, clinical translation must overcome key challenges, including editing precision, efficient retinal-targeted delivery, and long-term safety [115]. For RLBP1 deficiency, subretinal AAV8-hRLBP1 delivery accelerates dark adaptation in Rlbp1^−/−^ mice by restoring Müller cell–mediated intraretinal visual cycle function [116,117]. These findings highlight the importance of cell-type-specific expression in the design of gene therapies.

Metabolic pathway intervention is another major approach designed to bypass or compensate for defective retinoid metabolism. Examples include oral supplementation with 9-cis-retinyl acetate to supply the active chromophore, thereby circumventing RDH5 dysfunction directly [118]. Small molecules that enhance residual enzymatic activity or modulate retinoid flux are also under development, and targeting lipid homeostasis in the RPE may alleviate the toxic effects of abnormal retinyl ester accumulation [119].

Cellular replacement therapies, such as the transplantation of stem cell-derived RPE cells, are also being investigated to replace dysfunctional tissues. Although preclinical models support their feasibility, critical barriers remain, including the poor survival of transplanted cells in vivo, limited functional integration, and the risk of immune rejection [57]. Future strategies may require a combination of cell therapy with gene or metabolic interventions to achieve long-term benefits.

In summary, FA serves as a paradigm for understanding how defects in a single visual cycle enzyme or binding protein can lead to severe yet often treatable night blindness. Mutations in RDH5 and RLBP1 disrupt a common pathological pathway characterized by impaired 11cRAL regeneration and the buildup of toxic retinyl esters. The progression of targeted interventions, including gene, metabolic, and cellular therapies, not only offers a logical foundation for future treatments but also solidifies FA’s role as a key model for understanding the wider principles of retinoid metabolic dysfunction.

A comprehensive overview of chromophore-related retinal disorders, their molecular mechanisms, and translational therapeutic strategies is summarized in Table 2.

## 5. Therapeutic Limitations and Real-World Barriers in Visual-Cycle

Despite substantial advances in gene replacement, visual-cycle inhibition, antioxidant therapy, and chromophore supplementation, several real-world obstacles continue to constrain clinical translation of these approaches. First, small-molecule visual-cycle modulators—such as emixustat, MB-002, and isotretinoin derivatives—have successfully reduced bisretinoid accumulation in preclinical models, but they have not consistently translated into functional benefits in patients [120]. These issues largely stem from a mismatch between biochemical surrogate endpoints (e.g., lipofuscin or A2E levels) and actual visual outcomes, as well as from dose-dependent suppression of chromophore regeneration, which can cause nyctalopia or delayed dark adaptation. Moreover, the slow natural progression of diseases like STGD1 and AMD further complicates the demonstration of meaningful clinical improvement within the limited durations of typical clinical trials [124].

Gene therapy faces additional and substantial challenges. While RPE65 replacement has validated the concept of targeting the visual cycle, extending this success to larger genes remains difficult. For example, ABCA4, whose full coding sequence exceeds the packaging capacity of conventional AAV vectors, often requires dual- or triple-AAV systems that suffer from reduced transduction efficiency and increased complexity [56]. Pre-existing immunity to AAV, subretinal delivery-associated inflammation, and the technical demands of surgical delivery further restrict broad application. Additionally, the long-term durability of transgene expression remains uncertain, raising important questions about sustained efficacy and safety [125].

Oral small-molecule therapies also face pharmacokinetic and safety barriers. Many of these agents have limited ocular bioavailability and can produce systemic off-target effects. Over-suppression of chromophore regeneration may compromise visual sensitivity, especially under low-light conditions. In STGD1, several promising oral strategies are in development: for instance, deuterated vitamin A (gildeuretinol, ALK-001) slows the dimerization of vitamin A (thereby reducing toxic byproducts like A2E) without significantly impairing visual cycle function [126]. The TEASE-1 Phase 2 trial demonstrated a ~21% slowing in the growth rate of atrophic retinal lesions over two years, with good tolerability [127]. ALK-001 has also received Breakthrough Therapy and Orphan Drug designations from the FDA. Nonetheless, achieving sufficiently high concentrations in the retina without causing systemic toxicity remains a core challenge. Other agents—such as RBP4 antagonists (e.g., STG-001, Tinlarebant/LBS-008)—aim to slow retinoid flux, but dose optimization to balance efficacy and safety is still being refined [128,129].

Beyond these pharmacological challenges, economic, manufacturing, and regulatory hurdles present additional barriers. Gene and cell therapies are extremely costly, and scaling up GMP-grade vector or cell production is nontrivial. Regulatory pathways remain uncertain, particularly for genome editing, combinatorial treatments, and novel delivery platforms. The lack of validated surrogate biomarkers for efficacy further complicates trial design and endpoint selection, increasing development risk.

Long-term safety is another critical concern that slows clinical adoption. Persistent AAV expression may provoke delayed immune reactions [130], chronic chromophore supplementation could lead to retinoid toxicity [131], CRISPR-based editing carries potential off-target genomic risks [132], and the stability, integration, and immunocompatibility of transplanted iPSC-derived RPE cells remain incompletely characterized [133].

In summary, while therapeutic innovation in visual-cycle modulation is moving forward rapidly, there remain substantial mechanistic, biological, regulatory, and translational challenges. Overcoming these barriers will be essential to enable broad clinical adoption. Future research priorities should include developing more efficient and less immunogenic gene delivery vectors, optimizing small-molecule pharmacokinetics to maximize retinal exposure while minimizing systemic effects, establishing robust biomarkers for early efficacy assessment, and conducting long-term safety studies in clinically relevant models.

## 6. Conclusions

The classical visual cycle is a tightly regulated metabolic system essential for phototransduction, and its dysregulation drives a wide spectrum of inherited and acquired retinal degenerations. Accumulation of toxic retinoid intermediates—particularly all-trans-retinal and bisretinoids—triggers oxidative stress, RPE dysfunction, and progressive photoreceptor loss, integrating defects in RPE65, LRAT, RDHs, and ABCA4 into a common pathogenic axis. Although recent therapeutic advances, most notably RPE65 gene replacement, have validated components of this pathway as viable therapeutic targets, many fundamental biological questions remain unresolved. Key uncertainties include how visual-cycle enzymes coordinate retinoid trafficking under metabolic stress, how aldehyde overload intersects with proteostasis and inflammatory signaling, and how early biochemical disruptions can be detected before irreversible cellular damage occurs.

A major barrier to clinical translation is the continued absence of sensitive and predictive biomarkers capable of identifying early dysfunction or monitoring therapeutic response. Future progress will require the integration of multi-omics profiling, advanced structural and functional genomics, and high-fidelity retinal organoid or in vivo models to map how chromophore metabolism adapts—or fails to adapt—across disease stages. Recent evaluations of visual-cycle modulators and RBP4 antagonists underscore both the therapeutic potential and the difficulties of safely modulating retinoid flux in vivo. In parallel, the development of more efficient and scalable delivery platforms for large genes such as ABCA4, together with mutation-specific genome- and RNA-editing approaches, is expected to accelerate the implementation of precision-medicine strategies. The adoption of quantitative imaging tools and emerging machine-learning–based biomarkers will be critical for enabling earlier diagnosis, patient stratification, and genotype-informed trial design.

In summary, the classical visual cycle represents not only the biochemical foundation of vision but also a central metabolic axis whose perturbation underlies diverse retinal diseases. Advancing from disease attenuation toward durable vision preservation or restoration will depend on addressing unresolved mechanistic questions, establishing reliable early-stage biomarkers, and developing targeted, individualized therapeutic frameworks.

Although this review focuses on the RPE-based classical cycle, additional chromophore-renewal pathways—such as the Müller cell–mediated cone-specific visual cycle and the melanopsin regeneration pathway in intrinsically photosensitive retinal ganglion cells—constitute parallel “grand cycles” of visual function. Briefly acknowledging these systems highlights the broader landscape of photopigment regeneration and points to promising directions for future investigation.

## Figures and Tables

**Table 1:** Kinetic Characteristics of Key Enzymes and Transporters in the Classical Visual Cycle.

Enzyme/Transporter	Location	Primary Substrate(s)	Typical Km (approx.)	Cofactors/Requirements	Key Functional Notes
RPE65	RPE (ER membrane)	All-trans-retinyl esters	0.8–3 μM (for atRE)	Fe^2+^-dependent; requires LRAT-generated esters; membrane-associated	Rate-limiting isomerohydrolase; produces 11cROL [27].
LRAT	RPE (ER membrane)	atROL + phosphatidylcholine	2–7 μM (atROL)	Requires phosphatidylcholine; membrane-associated	Generates atRE substrate for RPE65; essential for cycle initiation; loss causes LCA; highly efficient in retinyl ester formation even at low [27].
RDH5	RPE	11cROL → 11cRAL	1–3 μM	NAD^+^-dependent	Main RPE enzyme for 11cRAL; reduced function causes FA [29,30].
RDH11	RPE	11cROL, atROL	2–5 μM	NADPH/NAD^+^ (dual)	Compensates for RDH5; detoxifies atRAL [29,30].
RDH8	Photoreceptor outer segment	atRAL → atROL	0.1–0.4 μM (very low)	NADPH-dependent	Rapid atRAL clearance in photoreceptors; prevents toxicity; loss ↑ A2E accumulation [20].
RDH12	Photoreceptor inner segment	atRAL → atROL	0.2–1 μM	NADPH-dependent	Inner-segment atRAL detoxification; mutations → LCA12/RP [20].
ABCA4	Photoreceptor disc membranes	A2PE	1–3 μM	Requires ATP hydrolysis	Clears NRPE/atRAL adducts; defects → A2E accumulation (STGD1) [38,39].
RLBP1/CRALBP	RPE & Müller cells	11cRAL, 11cROL	Kd < 20 nM	Requires binding pocket integrity; no enzymatic cofactors	Stabilizes and traffics 11-cis-retinoids; essential for efficient chromophore delivery; mutations → FA and delayed dark adaptation [31].

“→” represents the direction of enzymatic conversion.

**Table 2:** Retinal diseases associated with chromophore metabolic dysregulation, representative models, therapeutic strategies, and translational stage.

Disease	Key Mutated Genes	Toxic Metabolites	Core Pathogenic Mechanism	Representative Models	Current/Potential Therapies	Clinical Trial Status/Translational Stage
STGD1	ABCA4, RDH8	A2PE, A2E, atRAL	ABCA4 transport failure → bisretinoid accumulation → RPE apoptosis [45]	Abca4^−/−^, Abca4^−/−^Rdh8^−/−^ mice [39]	Visual-cycle modulators (emixustat) [120]; vitamin A deuteration (ALK-001) [121]; antioxidants (quercetin) [38]; gene therapy (dual-AAV, lentiviral, CRISPR) [122].	emixustat Phase 3; ALK-001 Phase 2; STG-001 Phase 2a; Tinlarebant Phase 3; quercetin Phase 3; Gene therapy preclinical.
dry AMD	RPE65, ABCA4 variants	A2E, lipofuscin, ROS	Age-dependent decline in visual cycle efficiency → A2E/atRAL buildup → RPE/Bruch’s dysfunction [63]	Light-induced, Abca4^−/−^ models, Abca4^−/−^Rdh8^−/−^ mice [41,70]	apocarotenoids (BIO203) [68,69], complement inhibitors (ongoing trials), PBM [75,76].	BIO203 preclinical/early clinical.
RP	LRAT, RPE65, RDH12 (among others)	atRAL excess	Chromophore crisis → ROS and oxidative stress → photoreceptor apoptosis [83]	Lrat^−/−^, Rpe65^−/−^, Rdh12^−/−^ mice [86,87]	RPE65 gene therapy (Luxturna) [123], chromophore replacement (9-cis-retinyl acetate), CRISPR-based editing [36].	RPE65 gene therapy FDA-approved; 9-cis-retinoids Phase 1/2; CRISPR editing in preclinical or early clinical.
LCA	RPE65, LRAT, RDH12	atRAL, retinyl ester imbalance	Block in chromophore regeneration → congenital blindness [91]	Rpe65^−/−^, Lrat^−/−^ mice [88,89]	RPE65 gene therapy (Luxturna) [123], gene therapy (in trials), chromophore replacement [100].	RPE65 therapy FDA-approved; LRAT/RDH12 gene therapies in early clinical or preclinical development.
FA	RDH5, RLBP1	Abnormal retinyl esters, impaired 11cRAL	Block in 11-cis-retinal regeneration → delayed dark adaptation [107,108]	Rdh5^−/−^, Rlbp1^−/−^ mice [109]	AAV8-RLBP1 gene therapy (preclinical) [116,117], chromophore replacement (9-cis-retinyl acetate) [118], iPSC-based RPE replacement [57].	AAV8-RLBP1 in preclinical stage; chromophore replacement Phase 1/2; iPSC-RPE early preclinical.

“→” represents the direction of enzymatic conversion.

## Data Availability

No new data were created or analyzed in this study.

## Abbreviations

Abbreviations and Full Terms in the Review.

**Abbreviation**	**Full Term**
A2E	*N*-retinylidene-*N*-retinylethanolamine
A2PE	*N*-retinylidene-*N*-retinyl-phosphatidylethanolamine
atRAL	All-trans-retinal
atROL	All-trans-retinol
ROS	Reactive oxygen species
11cRAL	11-cis-retinal
11cROL	11-cis-retinol
CRALBP	Cellular retinaldehyde-binding protein
CRBP	Cellular retinol-binding protein
ABCA4	ATP-binding cassette transporter 4
LRAT	Lecithin:retinol acyltransferase
RDH	Retinol dehydrogenase
RPE	Retinal pigment epithelium
RPE65	Retinal pigment epithelium-specific 65 kDa protein
RP	Retinitis pigmentosa
STGD1	Stargardt disease type 1
LCA	Leber congenital amaurosis
FA	Fundus albipunctatus
PBM	photobiomodulation
